# Feline Virome—A Review of Novel Enteric Viruses Detected in Cats

**DOI:** 10.3390/v11100908

**Published:** 2019-09-30

**Authors:** Barbara Di Martino, Federica Di Profio, Irene Melegari, Fulvio Marsilio

**Affiliations:** Laboratory of Infectious Diseases, Faculty of Veterinary Medicine, University of Teramo, 64100 Teramo, Italy; bdimartino@unite.it (B.D.M.); fdiprofio@unite.it (F.D.P.); imelegari@unite.it (I.M.)

**Keywords:** new enteric viruses, cats, etiology, epidemiology, pathogenesis, diagnosis

## Abstract

Recent advances in the diagnostic and metagenomic investigations of the feline enteric environment have allowed the identification of several novel viruses that have been associated with gastroenteritis in cats. In the last few years, noroviruses, kobuviruses, and novel parvoviruses have been repetitively detected in diarrheic cats as alone or in mixed infections with other pathogens, raising a number of questions, with particular regards to their pathogenic attitude and clinical impact. In the present article, the current available literature on novel potential feline enteric viruses is reviewed, providing a meaningful update on the etiology, epidemiologic, pathogenetic, clinical, and diagnostic aspects of the infections caused by these pathogens.

## 1. Introduction

Feline viral gastroenteritis is considered a common worldwide disease, especially in cats younger than one year of age living in high-density cat environments, such as catteries and shelters. The feline panleukopenia virus (FPV) [1,2,3], feline enteric coronavirus (FeCoV) [4,5], and feline leukemia virus (FeLV) [6] are the most important known viral causes of feline gastrointestinal disease, although various viral agents including astrovirus, adenovirus, rotavirus, and vesiviruses (feline calicivirus, FCV) have been sporadically detected in the stools of cats with enteritis signs by electron microscopy (EM) analysis [7,8,9,10]. 

Within the last decade, there has been a resurgence of interest for viral gastroenteritis that was sparked by the identification of novel viruses associated with diarrhea either alone or in mixed infections, occasionally resulting in more severe clinical signs. For many years, viral detection was restricted to a few specialized laboratories with EM equipment, and the etiology of a large portion of viral gastroenteric cases remained unknown. However, the introduction of diagnostic molecular tools mainly based on the use of broadly reactive primers, genus- or family-specific, targeting highly conserved genomic regions, increased the viral detection rate significantly, revealing that additional viruses, may be involved in the feline enteritis disease [11,12,13,14,15]. Furthermore, in recent years, using the advantages of metagenomic approaches for virus characterization and discovery, an unexpectedly high number of previously unknown viruses were detected in the feces of both healthy and diarrheic cats [16,17,18,19] (Table 1). 

Information on the epidemiology and genetic heterogeneity of these newly described viruses are still limited, and it is unclear whether these viruses may play a role as enteric pathogens of cats and to which extent they impact on feline health. 

The aim of this review is to provide an update on novel enteric viruses that have most recently been identified in association with enteritis signs in cats, focusing the main attention on feline norovirus, feline kobuvirus, and novel feline parvoviruses.

## 2. Feline Noroviruses 

Noroviruses (NoVs) are a major cause of epidemic gastroenteritis in children and adults. They cause nearly half of all gastroenteritis cases and > 90% of nonbacterial gastroenteritis epidemics worldwide [20]. NoVs have also been identified in several mammalian species, including cows, pigs, lion, dogs and cats, sea lions, bats, and harbor porpoises [11,21,22,23,24,25,26,27,28,29]. 

### 2.1. Etiology

NoVs are small non-enveloped viruses of 27–40 nm in diameter, belonging to the genus *Norovirus* in the family *Caliciviridae* [30]. 

The icosahedral capsid surrounds a 7.5–7.7 kb positive-sense single-stranded RNA genome covalently linked to Viral Protein g (VPg) at the 5′ end and polyadenylated at the 3′ end. NoV genome is organized into three Open Reading Frames (ORFs) [31]. ORF1 is translated as a large polyprotein which is co- and post-translationally cleaved by the virus-encoded protease (NS6) to release at least six mature non-structural (NS) proteins, including NS6. The other NS proteins include the viral RNA-dependent RNA polymerase (RdRp; NS7), VPg (NS5), the putative NTPase/RNA helicase (NS3), and NS1/2 and NS4, which have both been implicated in replication complex formation [32]. ORF2 encodes the major capsid protein (VP1), while ORF3 encodes the minor structural protein (VP2). 

The viral capsid is composed of 180 copies (90 dimers) of VP1, which consists of a shell (S) domain and two protruding (P1 and P2) domains [33]. The P2 domain extends above the viral surface and represents the most diverse region of the genome. The P2 domain is responsible for binding to histo-blood group antigens (HBGAs), which function as receptors or co-receptors on host cells [34], and it contains important determinants of antigenicity. Viral particles contain only a few copies of VP2, which are associated with the interior surface of the capsid formed by the S domain of VP1 [35,36].

Based on the full-length VP1 amino acid sequence, NoVs are classified into at least eight genogroups (GI to GVIII) and >40 genotypes [37]. Only GI, GII, GIV, and GVIII NoVs infect humans, with GII.4 strains that are the most prevalent worldwide [31]. NoVs detected in animals have been classified as GII (pigs) [38], GIII (small and large domestic ruminants) [39,40], GIV (lion, dog, cat) [11,25,26], GV (mice) [41], GVI (dog, cat) [42,43] and GVII [44]. 

The first evidence on the possible susceptibility of a member of the family *Felidae* to NoV infections was documented in 2006, in a four-week old lion (*Panthera leo*) died of severe hemorrhagic enteritis at the zoo of Pistoia, Italy [25]. Upon sequence analysis of the complete VP1 capsid protein, the lion NoV appeared to be genetically more related to human GIV NoVs (69.3%–70.1% amino acid (aa) identity in the capsid protein). Following strictly the outlines of Zheng’s classification [37], the novel NoV (strain lion/Pistoia/387/06/ITA) was classified as a distinct genotype (IV.2) (cut-off ≥ 85% pairwise aa identity intergenotypes), within the genogroup GIV (cut-off ≥ 55% pairwise aa identity intergenogroups); human GIV NoVs were classified as genotype 1. 

Shortly, after this first identification, additional evidence was found on the circulation of NoVs among carnivores. In 2007, a novel NoV strain (dog/Bari/170/07/ITA) was detected in a stool sample collected from a 60-day-old mixed-breed pup with diarrhea and vomiting, hospitalized at the Faculty of Veterinary Medicine of Bari (Italy) [26]. In the complete VP1, the highest sequence match was found to the lion GIV.2 strain (81.13% nucleotide [nt] and 90.1% aa identities), while the identity to human GIV.1 NoVs was 69.4%–68.2% aa (75.5%–74.0% nt).

Since then, by implementing the diagnostic algorithms of gastroenteritis cases with NoV-specific molecular assays, canine NoVs have been repeatedly detected [42,44,45,46,47], revealing a marked genetic diversity among the strains identified that allowed their classification into at least five genotypes from three distinct genogroups, i.e., GIV.2, GVI.1, GVI.2, GVI.3, and GVII [47].

Direct evidence on the circulation of NoVs in cats was obtained in 2012 in New York State [11], in which NoVs RNA was detected in the stools of six out of 14 (42.8%) 8–12-week-old cats with enteritis from a feline shelter. The full-length genomic sequence (7839 nt) of one feline NoV, CU081210E/ 2010/US, was determined. In the VP1, the novel virus displayed the highest aa identity to the GIV.2 NoV strain lion/Pistoia-387/06/IT (97.9%) and to the strain dog/Bari-170/07/IT (90.4%).

**Figure 1:**
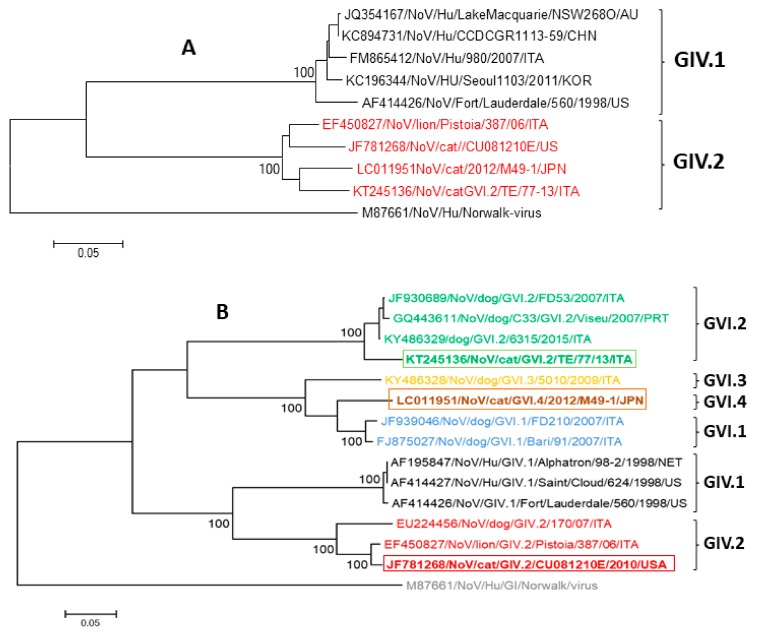
(**A**) Phylogenetic analysis based on the 750-nt sequence of the COOH terminus of the polymerase complex (RdRp) of GIV NoVs. The tree was generated using the neighbor-joining method and the Kimura two-parameter model. (**B**) Phylogenetic tree based on the full-length of the aa sequence of the VP1 protein of GIV and GVI NoVs, generated using the neighbor-joining method and Poisson correction. The rectangle delimits the VP1 sequences detected in cats.

More recently, two novel feline NoVs have been identified in Japan [43] and Italy [48], respectively. Based on sequence and phylogenetic analysis of the 3′ partial sequence of ORF1 spanning 750 nt, at the COOH terminus of the polymerase complex, Japanese (M49-1/2012/JPN) and Italian (TE/77-13/ITA) strains showed the highest identity to the GIV.2 NoVs (strains lion/Pistoia/387/06/ITA and cat/CU081210E/USA/2010) (91.0–93.0% nt and 97.0–98.0% aa) (Figure 1A). Interestingly, for both these novel feline NoVs, inconsistencies were observed between the RdRp- and capsid-based phylogeny, suggesting potential recombinant events. In the complete VP1 protein, the highest identities have been found to canine GVI NoVs. Based on Zheng’s criteria [37], the Japanese strain M49-1/2012/JPN was tentatively classified as novel genotype (75.5–81.6% pairwise aa identity intergenotypes) within the genogroup VI (GVI.4) [47], while the Italian feline strain TE/77-13/ITA was classified as a genotype 2 (GVI.2) together with canine NoVs [42,47] (93.0–94.0% pairwise aa identity intergenotypes) (Figure 1B).

Along with the accumulation of point mutations, recombination is a powerful mechanism strongly influencing the evolution and epidemiology of human NoVs [49]. Recombination observed in feline NoVs has also been described for canine NoVs [45,47]. In all the cases, the site of recombination was mapped to the highly conserved ORF1/ORF2 junction region. Accordingly, a definitive characterization of the strains circulating in carnivores should rely on the sequence analysis of either RdRp region of the ORF2 gene.

### 2.2. Epidemiology and Pathogenesis

The identification of the GIV.2 NoV strain lion/Pistoia-387/06/IT in the lion cub [25] also represents the first report describing the possible association of these newly discovered NoVs with clinical signs characterized by severe hemorrhagic enteritis. The lion tested negative for common feline and canine viral pathogens, but it was found co-infected with toxigenic clostridia. The detection of NoV in a lion raised questions on the role of the carnivores in the epidemiology of this virus. Accordingly, in order to acquire additional information, an enzyme-linked immunosorbent assay (ELISA) based on the recombinant VP1 capsid protein of the lion NoV strain expressed by baculovirus system was developed and employed to screen a collection of cat and dog sera [50]. IgG antibodies against GIV.2 NoVs were detected in 16.1% of cats and in 4.8% of dogs, providing the first serological evidence on the circulation of these NoVs in domestic carnivores. In the molecular investigation performed by Pinto et al. [11], the RNA of GIV.2 NoVs was detected in 6/14 samples collected from kittens with enteritis housed in a shelter in New York State [11]. Interestingly, upon sequence analysis of the ORF2 gene, the six NoVs were identical to each other, suggesting the spread of a unique strain within the shelter. Subsequent molecular studies reported detection rates of GIV.2 NoVs in cats ranging from 1.2% in Japan [51] to 2.8% in Brazil [52] and to 6.2% in Italy [48], either alone or in mixed infections with other viral pathogens such as FPV, FeCoV, feline calicivirus, and feline kobuvirus [48,52].

Except for one study in which GIV.2 NoV like-sequences were identified by the metagenomic approach analyzing pooled fecal samples from clinically healthy cats [17], to date NoVs have been found only in the diarrheic ones. In a molecular survey conducted in Italy [48], NoVs were detected in animals with enteric signs with a prevalence rate of 6.2%, whilst they were not identified in samples collected from healthy cats used as the control study group. Furthermore, experimental inoculation of specific pathogen-free cats with the feline GVI.1 strain JPN/2012/M49 could induce enteritis signs, diarrhea and vomiting [43,53]. Taken together, these findings seem to indicate a possible role of these NoVs as feline enteric pathogens, although their pathogenic role in cats should be confirmed in larger epidemiological surveys and in experimental infections with other NoV genotypes.

### 2.3. Diagnosis

The broadly reactive primers p289–p290 [54] targeting the highly conserved motifs (DYSKWDST and YGDD) of the RdRp region of caliciviruses and the norovirus-specific primers pair JV12Y/JV13I [55] amplifying the same region, have been both successfully employed to detect NoVs in carnivores [25,26,45,47,48]. Furthermore, a specific RT-PCR strategy targeting the RdRp region has been developed based on the alignments of the cat and lion NoV sequences [11]. However, given the high genetic heterogeneity of carnivores NoVs and the frequency of recombinant events, a more detailed characterization should rely on the determination of larger genomic sequence spanning the RdRp region and the complete ORF2 gene. The strategies mainly used to determine the sequence of ~3.4-kb fragment of the NoV genome (the 3′ end of ORF1, the full-length ORF2, ORF3, and the noncoding region through the poly-A tail) are based on the application of 3′ RACE (Rapid amplification of cDNA ends) protocol previously described by Scotto–Lavino et al. [56]. However, also the specific primer sets targeting the complete ORF2 gene [11] of feline NoVs have been successfully used to acquire additional genetic information (Table 2).

Except for the murine NoV [57], a reproducible system culture allowing the routine replication of in vitro NoVs is still not available. To date, there are two published systems (B cell line and stem cell-derived enteroids) [58,59] supporting replication of human NoVs, but the levels of replication are not sufficient for the generation of highly purified virus stocks or the development of culture-based quantification assays [60].

Serological studies of NoVs necessarily rely on the expression of synthetic antigens, and the baculovirus system appears to be particularly adequate since the baculovirus-expressed full-length VP1 of NoVs is assembled into virus-like particles (VLPs) morphologically and antigenically indistinguishable from wild-type NoVs [61]. ELISAs based on VLPs have successfully been used to gather information on the epidemiology of NoVs in humans and animals [50,62,63,64,65,66]. To date, only one seroprevalence investigation was performed in the cat population in Italy [50] by using an ELISA assay based on the VLPs of the GIV.2 NoV strain lion/Pistoia-387/06/IT [25].

## 3. Feline Kobuvirus

Feline kobuvirus (FeKoV) is a newly discovered virus, belonging to the genus *Kobuvirus*, within the family *Picornaviridae* [67].

Human Aichi virus (AiV) strain A846/88, the prototype strain of the genus *Kobuvirus*, was first discovered in 1989 as the cause of oyster-associated nonbacterial gastroenteritis in humans in Aichi Prefecture, Japan [68]. Since then, several investigations have revealed that AiVs are involved in 0.9–4.1% of sporadic cases of pediatric gastroenteritis [69,70,71,72].

Recently, novel kobuviruses genetically closely related to human AiVs have been identified in domestic and wild carnivores including dogs [73,74], cats [13], red foxes [75], golden jackal, side-striped jackal, spotted hyena [76], and wolves [77].

### 3.1. Etiology

**Figure 2:**
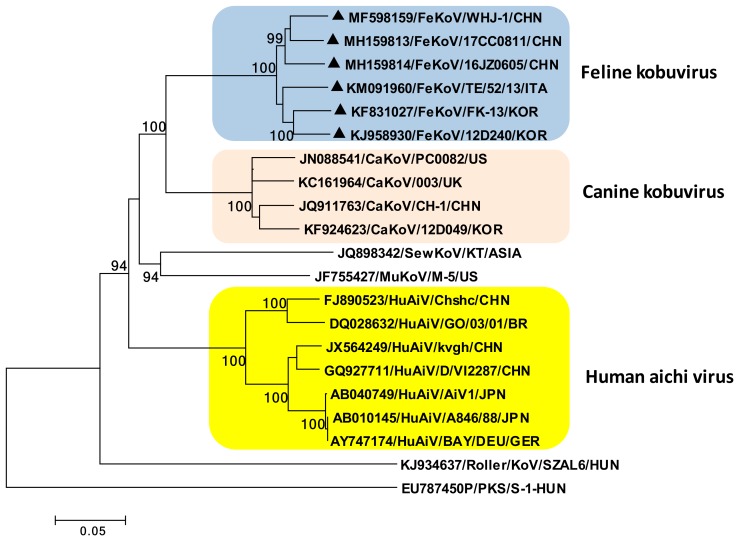
Phylogenetic tree based on the full-length genomic sequence of the species *Aichivirus A*. The tree was generated using the neighbor-joining method and the Kimura two-parameter model supplying a statistical support with bootstrapping of 1000 replicates. Black triangles indicate the FeKoV sequences. Abbreviations: FeKoV, feline kobuvirus; CaKoV, canine kobuvirus; SewKoV, sewage kobuvirus; MuKoV, murine kobuvirus sewage; HuAiV, human aichi virus; PKS, porcine kobuvirus.

Kobuviruses are non-enveloped, icosahedral viruses of approximately 27–30 nm in diameter. Based on the genomic organization and sequence similarities, the genus *Kobuvirus* currently includes six officially recognized species: *Aichivirus A* (formerly *Aichi virus*), *Aichivirus B* (formerly *Bovine kobuvirus*), *Aichivirus C* (porcine kobuvirus), *Aichivirus D* (kagovirus 1), *Aichivirus E* (rabbit kobuvirus) and *Aichivirus F* (bat kobuvirus 1). Feline kobuvirus (FeKoV) is classified within the species *Aichivirus A*, along with the human AiV, canine kobuvirus (CaKoV), murine kobuvirus (MuKoV), Kathmandu sewage kobuvirus, and roller kobuvirus (Figure 2) [78,79].

FeKoVs were firstly discovered in diarrheic cats in South Korea [13]. Sequence analysis of the partial 3D region (RNA-dependent RNA polymerase) revealed that feline strains shared the highest identities with strains previously detected in dogs (82.1% nt and 92.1% aa identities) [73,74,80,81], in rodents (79.9% nt and 89.4% aa) [82], and in humans (80.4% nt and 88.7% aa) [68]. Subsequently, the complete genome of six FeKoV strains (FK-13, 12D240, TE/52/13/ITA, WHJ-1, 16JZ0605, and 17CC0811) detected in South Korea, Italy and China [83,84,85,86,87] were characterized, confirming that FeKoV strains represent a distinct group of kobuviruses within the species *Aichivirus A*.

**Figure 3:**
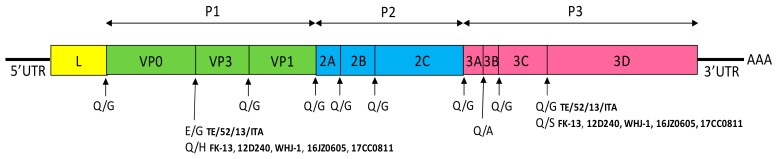
Schematic genome organization of FeKoV. The 5′ UTR, 3′ UTR, and the ORF (boxes) are indicated. The different predicted cleavage sites for the six complete genome sequences available on GenBank (TE/52/13/ITA, FK-13, 12D240, WHJ-1, 16JZ0605, 17CC0811) are shown below each gene border.

FeKoV genome is polyadenylated, single-stranded, positive-sense RNA of 8.2 kb in length, that contains 5′ untranslated region (UTR) of 646–717 nucleotides (nt), one single open reading frame (ORF) of 7308–7467 nt and 3′ UTR of 241–244 nt. The unique ORF encodes a single large polyprotein of 2436–2437 amino acids (aa) that undergoes protease processing to yield a leader protein (L), three structural capsid proteins (VP0, VP1 and VP3) and seven non-structural proteins (NSPs) (2A, 2B, 2C, 3A, 3B, 3C, 3D). The FeKoV genome, like that of other kobuviruses, could be divided into three functional regions: the P1 encoding the structural proteins VP0, VP1 and VP3, P2 encoding the NSPs 2A-2C and P3 encoding the NSPs 3A–3D [88,89]. Putative cleavage sites of the polyprotein all contain primary Q/G amino acid residues, except for E/G and Q/A between VP0/VP3 and 3A/3B, respectively in the Italian TE/52/13/ITA strain, and Q/H, Q/A, Q/S between VP0/VP3, 3A/3B, and 3C/3D, respectively, in the Korean and Chinese strains (Figure 3).

Genetic distance calculations based on the analysis of the full-length polyprotein of the six FeKoV strains to date available on GenBank database revealed a high identity to each other (92.9%–93.4% nt and 96.8%–98.5% aa), that is consistent with the genetic similarities found for human AiVs and CaKoVs. More recently, further information on the genetic diversity of these group of kobuviruses has been obtained by Niu et al. [87] in a molecular survey performed in Northeast China by analyzing the structural VP1 gene of eight FeKoV strains. VP1 protein is the most exposed and immunodominant part of the picornavirus capsid proteins, and it is also the most variable structural protein among the six kobuvirus species [70]. Upon sequence analysis based on the complete VP1 sequences, the Chinese FeKoVs formed a tight group, distinct from other strains previously detected in South Korea [90] and Italy [85]. Furthermore, three identical aa substitutions (182, 235, 241 are the aa positions referred to the VP1 protein) were present at the C-terminal of the VP1 protein of all FeKoV strains detected in China. Accordingly, it has been hypothesized that VP1 could be employed as a molecular marker to acquire information on the geographical origin of the feline strains detected [87].

### 3.2. Epidemiology and Pathogenesis

The first evidence on the susceptibility of cats to kobuvirus infections was obtained in the UK [81] by a serological screening based on of the prototype human AiV strain A846/88 [68] as an antigen. Out of the 97 sera tested, 69.9% (67/97) contained specific IgG antibodies, suggesting a high circulation of AiV-like viruses in the feline population investigated. More exact virologic information on the seropositivity found in cats was documented in the same year [13] in South Korea by assessing a collection of stool samples from cats molecularly with diarrhea that revealed the presence of FeKoV RNA in six specimens with an overall prevalence of 15.4% (6/39). After this first direct identification, additional four molecular studies [85,86,87,90] confirmed the active circulation of these novel kobuviruses in cats either in Asiatic or European countries, with rates ranging from 13.5% to 28.8%.

Although, the relationship between infections and gastroenteritis in cats is far from elucidated and still requires further epidemiological and experimental studies, the frequency of detection of FeKoV has been found to differ significantly between symptomatic and asymptomatic cats [85,86]. In a molecular survey performed in Italy [85], all FeKoV positive samples (13.5%, 5/37) were collected from cats with signs of enteritis; none of the healthy cats were found positive (0/46) (Table 3).

In most cases, FeKoV-infected cats were also co-infected by other viral pathogens like FPV, FeCoV, feline NoV, and feline bocavirus (FBoV) [48,86,87]. Furthermore, kittens under six months of age seem more susceptible to infection, likely because of an inefficient immune response or other intrinsic age-related factors [13,90]. Finally, the positive rate of animals from shelters appeared higher than that of cats from private veterinary clinics [87,90], suggesting that animal promiscuity in enclosed settings enhances the circulation of pathogens, including enteric viruses like FeKoV that are commonly shed in the feces of infected animals and transmitted by the fecal-oral route [70].

### 3.3. Diagnosis

Conventional RT-PCR is the primary assay for the detection of FeKoVs. The first identification of FeKoVs RNA was obtained by using the primers set 10f and 10r [91] targeting a 631 bp fragment of the 3D region highly conserved between bovine kobuvirus prototype strain U1 and human AiV strain A846/88 [68]. Currently, the RT-PCR strategy widely used to screen feline stool samples is performed by using generic kobuvirus primers (UNIV-kobu-F (Forward, 5′-TGGAYTACAAG(/R)TGTTTTGA-3′ and UNIV-kobu-R (Reverse, 5′-ATGTTGTTRATGATGGTGTTGA-3′) amplifying a region of 216 bp of the 3D region and able to recognize all the members of the genus *Kobuviruses* [92]. These broadly reactive primers are successfully used to detect kobuviruses in cats and in other animal species [75,77,80,85].

Replication in vitro of kobuviruses has been demonstrated only for AiV strain A846/88 [68] and bovine kobuvirus strain U-1 [91], determining both a distinct cytopathic effect on BS-C-1 and Vero cells, respectively. By converse, in spite of several attempts on different cell lines, replication of FeKoV in cell cultures has never been successful [86].

An ELISA and an immunofluorescence assay have been set up using the human AiV strain A846/88-infected cells as antigen. These assays were successfully used to assess the exposure of domestic carnivores to kobuviruses [81]. However, considering the extent of the genetic heterogeneity of kobuviruses, generation of synthetic antigens based on each capsid genotype (CaKoV and FeKoV) would be necessary.

## 4. Feline Parvoviruses

Parvoviruses (family *Parvoviridae*) are non-enveloped icosahedral viruses of 22–25 nm with a positive-sense single-stranded (ss) DNA genome of 4.5–5.5 kb with complex hairpin structures at the 5′ and 3′ ends, and it encodes three or fpur proteins; non-structural (NS) 1, nucleoprotein (NP) 1, and viral protein (VP) 1 and VP2 [93]. The *Parvoviridae* family is divided into two subfamilies, *Parvovirinae* and *Densovirinae* [94], infecting vertebrates and arthropods, respectively. In the *Parvovirinae* subfamily is enclosed the genera *Bocaparvovirus* and *Protoparvovirus*, whose members have been identified in a wide range of mammalian species, including domestic carnivores.

Feline parvovirus (FPV), currently classified within the species *Carnivore protoparvovirus 1* (*Protoparvovirus* genus), causes feline panleukopenia, the oldest known viral disease of cats [1], characterized by severe panleukopenia and enteritis in cats [2,3] and cerebellar ataxia in kittens [95]. Infection is highly contagious, often associated with high mortality and morbidity [96].

More recently, novel parvoviruses belonging to the genera *Bocaparvovirus* and *Protoparvovirus* have been identified in cats [12,14,16,17] (Table 4), raising several questions on their possible association with clinical disease. Interestingly, these viruses have been detected either in the feline intestinal content but also in respiratory [12,14], blood, urinary, and kidney samples [12].

### 4.1. Feline Bocaparvoviruses

Bocaparvoviruses (BoVs) have been detected in a number of mammalian species including humans and non-human primates [97,98,99,100], pigs [101,102], California sea lions [27], bats [103], rabbits [104], minks [105], rats [106], pine martens [107], dogs [12,108,109], and cats [12,16,17].

Common structural features of the bocaparvoviruses include the approximately 5.5 kb ssDNA genome and two main ORFs coding the NS1 (ORF1) and the capsid protein precursor VP1/VP2 (ORF2). A third additional ORF (ORF3), located between ORF1 and ORF2, encodes the NP1 [110], a highly phosphorylated protein that is not similar to proteins of other parvoviruses and appears essential for virus replication, although its function is still poorly understood [111,112]. Current ICTV guidelines [113] use 85% aa identity along the NS1 protein to establish bocaparvovirus species. Based on this criterion, the genus *Bocaparvovirus* is classified into twenty-three officially recognized species, with at least five species detected in domestic carnivores and designated *Carnivore bocaparvovirus* 1–5; a sixth species (*Carnivore bocaparvovirus 6*) includes bocaparvoviruses detected in minks [105]. Feline bocaparvoviruses (FBoVs) to date identified in domestic cats have been classified within the species *Carnivore bocaparvovirus 3* [12], *Carnivore bocaparvovirus 4* [16], and *Carnivore bocaparvovirus 5* [17].

FBoV DNA was firstly identified by assessing fecal, nasal, urine, kidney, and blood samples collected from stray cats during a large molecular survey conducted in Hong Kong [12]. The near-complete genomic sequences (5179–5331 nt) were obtained for two strains (HK797F and HK875F) of enteric origin, whilst a third strain (HK797U) was detected in one urine sample. Upon sequence analysis, the three FBoV genomes displayed the highest identities (58.6–59.7% aa) to the minute virus of canines (MVC) [108]. Following strict classification criteria [113], the newly discovered feline parvoviruses have been classified within a novel species named *Carnivore bocaparvovirus 3*. Subsequently, by metagenomics analysis of feces collected from a healthy cat in Portugal [16], an additional near-complete bocavirus genome was detected (strain POR1). The strain POR1 showed in the complete NS1 a low amino acids identity (58%) with the three FBoVs previously detected in stray cats in Honk Kong [12]. Accordingly, the FBoV2-POR1 has been classified as a distinctly designed species *Carnivore bocaparvovirus 4* [16]. The third group of bocavirus, FBoV3, genetically diverse from FBoVs and FBoV2 strains, and then classified as an additional novel species, *Carnivore bocaparvovirus 5*, has been detected in California [17] by next-generation sequencing (NGS), assessing fecal pools collected from 25 cats of a shelter. Since then, FBoV2 infections have been reported in Japan [114], both FBoV and FBoV2 strains were detected in Northeast China, and more recently a FBoV3 strain was identified in Canada [19,115,116]. These findings suggest that genetically diverse FBoVs widely circulate in cats in different geographical areas. However, it is not yet known whether this genetic diversity may affect the biological properties of the various FBoV species. Bocaparvoviruses infections have been identified as a possible cause of enteric, respiratory, reproductive/neonatal, and neurological disease. Human bocaparvoviruses have been detected in children with respiratory infections and gastroenteritis, but the pathogenic potential is still uncertain since they were also detected in healthy children and frequently in co-infection with other pathogens [97,117,118,119,120,121,122]. Animal bocaparvoviruses have been detected in both healthy and symptomatic animals, mostly young, with the respiratory, gastrointestinal, and reproductive disease [123]. In dogs, it has been confirmed that the infection is linked to gastroenteritis [124]. Furthermore, the detection in extra-intestinal sites such as respiratory tract, liver, and blood, raises questions on the possibility that these viruses could cause systemic infections [109,125].

The pathogenicity of FBoV has not been experimentally assessed and it is not clear if this virus may play a role in enteric or extra-enteric diseases in cats. FBoV DNA was first identified in multiple tissues, specifically in 26 (7.2%) of 363 fecal samples, six (1.6%) of 364 urine samples, three (0.8%) of 361 blood samples, one (0.3%) of 364 nasal samples, and one (2.0%) of 51 kidney samples collected from a total of 364 stray cats in Hong Kong, suggesting a wide tissue tropism [12]. After this investigation, these viruses were repetitively detected in fecal samples of healthy cats [16,17]. In a molecular study conducted in Japan [114], FBoV2 DNA was identified in four of 48 (8.3%) rectal swabs collected from healthy cats and in six of 53 (11.32%) animals with gastroenteritis, although no significant association was found between FBoV2 infection and clinical disease [114]. In a more recent investigation performed in China [115], out of 36 fecal samples assessed, FBoV DNA has been found in one of 13 cats with severe enteritis, while it was not detected in asymptomatic animals. In a second molecular survey conducted in China [116] a significant association was observed between FBoV infection and diarrhea. In most cases, FBoV-infected cats were also co-infected by other viral pathogens such as rotavirus, astrovirus, bocavirus, feline sakobuvirus, and picobirnavirus [16,114]. These findings seem to indicate that FBoV are a common component of the feline fecal virome. The implementation of the diagnostic algorithm of feline enteritis with FBoVs specific tools could help to gain insight on the enteropathogenic role of these viruses. Although replication in vitro has been demonstrated for various human and animal bocaparvoviruses [126,127,128,129], attempts to culture FBoV in different cell lines were unsuccessful [12]. Detection of FBoVs could be obtained by conventional PCR. A consensus primer set targeting a 141 bp fragment of the NS1 gene has been used for the first identification of FBoV DNA [12]. Subsequently, specific PCR strategies able to detect each of the three different FBoV types were developed, employing primer sets targeting regions of FBoV and FBoV2 NS1 genes [12,16], and of FBoV3 VP1 gene [17] (Table 5).

### 4.2. Feline Protoparvoviruses

The species *Carnivore protoparvovirus 1* (genus *Protoparvovirus*) includes genetically and antigenically related viruses such as FPV, canine parvovirus (CPV-2), mink enteritis virus (MEV), and raccoon parvovirus (RaPV) [130], causing all serious diseases, especially in young animals.

FPV, isolated for the first time in 1965 [131], has been recognized as cause of disease in cats since the beginning of the twentieth century [1]. Given the need to replicate in mitotically active tissue such as bone marrow, lymphoid tissues, and intestinal crypt cells, FPV is responsible of systemic infections characterized by severe panleukopenia and enteritis. Furthermore, the intrauterine or perinatal infection could result in cerebellar hypoplasia with ataxia and intention tremor of kittens [95]. However, it has been demonstrated that several feline panleukopenia outbreaks in cats are not caused by classical FPV strains, but by variants of the CPV-2 [132,133,134]. CPV-2 was first identified in dogs in the late 1970s in Europe and North America, mainly associated with serious hemorrhagic gastroenteritis and myocarditis in puppies [135] and likely arisen from FPV after adaptation in an unknown wild-carnivore species [133]. Shortly after its identification, the original CPV-2 type, which cannot infect cats, was replaced by three variants, named CPV-2a, CPV-2b, and CPV-2c [136,137,138], that have regained the ability to replicate in vivo also in the feline host [139,140].

In 2017, a novel protoparvovirus distantly related to FPV and CPV-2 was identified in cats, by assessing respiratory specimens collected from cats with or without signs of upper respiratory tract disease (URTD) and stool samples from diarrheic animals [14]. Sequence analysis of the nearly complete VP2 coding region of three feline strains (ITA/2012/TE109, ITA/2015/BA509, and ITA/2017/BA291) [14] revealed that the viruses displayed > 99.9% nt sequence identity to a novel canine parvovirus, designated canine bufavirus (CBuV) [141], firstly detected in a litter of five-month-old puppies during an Italian outbreak of canine infectious respiratory disease (CIRD). In the NS1 protein, CBuV displayed low aa (19.3%–51.4%) identity compared to members of the species *Carnivore protoparvovirus 1*, while the closest relatives to CBuV were protoparvoviruses identified (47.2%–51.4% aa identity in NS1) in human and non-human primates, commonly termed as bufaviruses (BuVs) [142,143,144]. On the basis of the classification criteria established by ICTV (>85% aa identity in the NS1 protein) [130], the newly discovered canine and feline protoparvoviruses could be considered members of a new species within the genus *Protoparvovirus*, for which the name “*Carnivore protoparvovirus 2*” has been proposed [14,141]. More recently, by using a broadly reactive primer pair 165F (5-CTGGTTTAATCCAGCAGACT-3′) and 371R (5′-TGAAGACCA AGGTAGTAG GT-3′) [141], which target a 207-bp region of the VP2 encoding gene of feline and canine BuVs, viruses genetically closest to carnivore protoparvoviruses 2, have been also identified in fecal samples collected from foxes and wolves in Italy [145].

Whether these protoparvoviruses are associated with any disease in cats is not yet known. Until now, BuVs have been identified in humans and recently in wild carnivores (wolves and foxes), exclusively in the enteric tract [145,146], but other investigations in dogs [141,147], monkeys [144], shrews [148], and in sea otters [149], suggest possible extraintestinal and systemic BuV infections. Molecular screening of feline fecal samples and respiratory swabs revealed that the virus was more common in nasal and oropharyngeal samples (10.2%) than in enteric specimens (2.2%). Furthermore, higher positivity rates were observed in cats with respiratory signs (16.4%, 29/176) than in asymptomatic animals (8.7%, 17/196) [14]. Similar results were obtained when analyzing fecal and respiratory samples of dogs [141]. More recently, canine BuVs were detected in serum specimens from dogs in China. Of interest, the positive sera were collected from animals showing signs of CIRD [147]. Accordingly, a preferential tropism for the respiratory tract for these viruses has been hypothesized, although the identification of these viruses in enteric tract of domestic carnivores and more recently also in wild canids [145] should be further investigated in order to exclude their enteropathogenic role and the possibility that fecal shedding of BuVs represents a strategy of virus persistence in animal populations.

## 5. Other Viruses

In addition to noroviruses, kobuviruses, and parvoviruses, other viruses such as astroviruses, rotaviruses, novel circular replication-associated protein-encoding single-stranded (CRESS) DNA viruses [15], and Lyon-IARC polyomaviruses (LIPyVs) [19], are suspected of having the ability to cause enteric disease in cats. Astroviruses (AstVs), family *Astroviridae*, are small, non-enveloped, spherical viruses of approximately 28–30 nm in diameter, with a positive single-stranded RNA genome of 6.8–7.3 kb in length organized in three ORFs (ORF1a, ORF1b, and ORF2) and a poly A tail at the 3′ end [150]. AstVs have been identified in human beings and in a variety of terrestrial and marine mammals, as well as in several avian species, and they are classified into two genera, *Mamastrovirus* (AstVs of mammals) and *Avastrovirus* (AstVs of avians) [151]. AstVs are currently considered as one of the most common viruses associated with either mild or severe gastroenteritis in humans, mainly in young children and immunodeficient patients [152]. Feline astrovirus (FAstV) was first identified by EM from the stool of a domestic kitten with diarrhea in the USA [7]. Subsequent EM-based investigations revealed the presence of FAstV either in the stools of diarrheic or healthy cats [9,153]. In the last few years, molecular tools have allowed characterizing in more detail the FAstVs infecting cats demonstrating the circulation of strains genetically closest to each other that have been classified within the species *Mamastrovirus 2*, formerly known as feline astrovirus. Sequence analysis of the complete ORF2 gene revealed that the feline strains clustered within two distinct groups that have been recently proposed as different genotypes (group 1 and 2) [154]. Furthermore, mamastroviruses more genetically closest in the RdRp region to AstVs previously found in foxes [155] have occasionally been identified in fecal samples collected from healthy cats [17,156].

To date, FAstVs have been detected in stool samples of cats in UK [153], Australia [9], New Zealand [8], Italy [157], Hong Kong [158], South Korea [159], Portugal [16], Northeast China [154], and the USA [17,156,160] with prevalence rates ranging from 4.8% to 28.6%. The role of AstV as enteric pathogen in cats still remains unclear, although experimental infection using the FAstV strain Bristol [153] in specific pathogen–free (SPF) kittens induced enteritis and viral shedding. Furthermore, natural infection has been described in domestic cats with diarrhea either alone or in mixed infections mainly with FPV [7,153,154,157,159].

Rotaviruses (RVs) have been recognized as a major cause of human acute gastroenteritis since 1973 [161]. They primarily affect young children, accounting for almost 40% of hospital admissions for diarrhea and 200000 deaths worldwide [162]. RVs (genus *Rotavirus*, family *Reoviridae*) are characterized by a 70–75 nm non-enveloped multi-layered virion with 11-segmented double-stranded RNA encoding six structural proteins (VP1–VP4, VP6, and VP7) and other five or six NSPs (e.g., NSP1–NSP5/NSP6) [162]. To date, nine rotavirus species (RVA to RVI) have been recognized [163], and a tentative tenth species (RVJ) has been described [164]. Among these, RVA to RVC, RVE, RVH, and RVI are known to infect mammals, with RVA being the most prevalent [164]. The genetic variability of the VP4 and VP7-encoding genes determines the binary RVA genotype classification system [165]. Currently, 27 G (VP7) and 37 P genotypes (VP4) of RVA have been described in mammals and avian species [166,167]. First evidence on the susceptibility of cats to feline rotavirus (FRV) infection was obtained serologically in 1978 by McNulty et al. [168]. Subsequent experimental infections gave contrasting results, with some showing an association between rotavirus and reduced fecal quality as increased water content and not optimal conformation of feces [169], while others failed to give infection with evident clinical signs [9,170]. Early epidemiological studies on rotavirus infection in cats has been conducted by serological assay and EM analysis. The serological studies reported prevalence ranging from 3.5% [171] to 100% [172], while EM revealed rates from 5% [9] to 6% [173] either in diarrheic or healthy animals. In a large molecular survey performed in the UK, FRVs RNA has been detected with an overall prevalence of 3.0% (57/1727); statistical associations with diarrhea or age have not been found [174]. Sequence analysis of the strains identified in cats showed the highest genetic correlation with RVs belonging to the A group [174]. More recently, an I group rotavirus was detected in the feces of a diarrheic seventh-month old indoor cats [18]. Phylogenetic analyses revealed that rotavirus I strain *Felis catus* shared a monophyletic root, being most closely related to the two currently known rotavirus species I strain detected in the feces of two sheltered dogs in Hungary [175]. Overall, FRVs are currently considered to play a minor role in clinical disease and are not routinely screened in diarrheic cases in small-animal veterinary practices [174]. However, whether and to what extent FRVs may impact feline health, deserves further study.

Mayor CRESS DNA viruses detected in animals are represented by circoviruses and cycloviruses belonging to the *Circoviridae* family [176]. The susceptibility of carnivores to circoviruses infections have been previously demonstrated in domestic and wild canids [177,178]. In dogs, these viruses have been found in association with clinical disease characterized by hemorrhagic gastroenteritis, severe necrotizing vasculitis, and granulomatous lymphadenitis [179,180]. By converse, information on the epidemiology of circoviruses in the feline host is still limited. The identification of CRESS-DNA genomes in stool samples has been reported only on two occasions [15,17]. By metagenomic sequencing, the complete genome of a cyclovirus strain (CyCVs-FD) was acquired assessing pooled fecal samples collected from clinically normal cats in a shelter in Davis, California [17]. More recently, by molecular screening of twenty stool samples collected from diarrheic and healthy cats from a private cattery in Japan [15], viral DNA was detected in 71.4% (10/14) of animals with enteritis signs and in 50% (3/6) of asymptomatic cats. Full-genome sequence analysis of four strains revealed that these novel CRESS-DNA genomes, called feline stool associated circular viruses (FeSCVs), clustered within the family *Circoviridae*, but into a distinct clade to that of circovirus and cyclovirus. Further investigations aimed to acquire information on the genetic features, epidemiology and pathogenesis of these viruses are needed. Polyomaviruses (PyVs), belonging to the *Polyomaviridae* family, are non-enveloped double-stranded DNA viruses with a circular genome of approximately 5.0 kb in length. PyVs DNA have been occasionally identified in fish, birds, rodents, and primates [181,182]. Interestingly, novel PyVs have been recently found in cat fecal samples during a diarrhea outbreak in Canada by using a metagenomic approach [19]. Upon sequence analysis, the feline PyVs revealed the highest identity (97.0%) to members of the genus *Alphapolyomavirus* detected in saliva and skin samples of human origin [183] and provisionally named Lyon IARC PyVs (LIPyVs). The etiologic role of LIPyV in feline diarrhea should be investigated as well as its zoonotic potential.

## 6. Discussion and Conclusions

In the last few years, a number of viruses have been found in association with enteric disease in cats. Many of them have been discovered serendipitously, using advanced molecular techniques to screen feline stool samples. The epidemiology of these newly discovered viruses is still largely unexplored, but several pieces of evidence suggest their possible role as primary causative pathogens or synergistic agents in feline gastrointestinal disease. In order to obtain a complete picture, each novel enteric virus should be included in the panel of pathogens for routine testing of cases of feline enteritis. Furthermore, large structured epidemiological studies and experimental infections might help to clarify any possible association with enteric diseases.

Interestingly, some of the viruses considered in this review have also been identified in the canine fecal virome, suggesting the possibility of inter-species circulation between the two carnivore species. Cats and dogs may harbor NoVs of the same genogroups and genotypes, GIV.2 and GVI.2 [11,26,48]. Binding of GVI.2 and GVI NoVs in dog tissues has been demonstrated to be mediated by the presence of the H and A antigens of the histo-blood group antigen (HBGA) family [184]. Accordingly, it has been hypothesized that dogs and cats share a similar pattern of HBGAs as the attachment factor for NoV infections [48]. Meanwhile, novel carnivore protoparvoviruses identical to each other in their capsid gene (>99.9% nt identity) have been found in stool and respiratory samples either in cats or dogs [14,141]. Since a few aa mutations in the VP2 can modify the host range of FPV and CPV-2, it has been hypothesized that the novel carnivore protoparvovirus 2 has recently crossed the species barrier from a yet unidentified source, with a recent bottleneck event in the evolution of this virus in domestic carnivores [141].

The global distribution of cats and their close contacts with humans represents an additional reason to better understand the composition of their enteric virome [16]. Historical evidence suggest that some feline viruses are potentially zoonotic. Infection of young children by rotavirus strains of feline origin has been documented in Italy [185] and more recently, in Germany [186]. The discovery of GIV.2 NoVs in cats and dogs genetically closest related to human GIV.1 NoVs has raised public health concerns about potential interspecies transmission between humans and pets. This eventuality has been demonstrated in a serosurvey performed in Italy on a collection of human serum samples, in which specific IgG antibodies against VLPs based on the lion GIV.2 NoV [25] have been detected with prevalence ranging from 6.8% to 15.1% among different age groups [64]. Furthermore, in a study conducted in Portugal [187], the presence of antibodies to GVI.2 NoV were found in 22.3% of the veterinarians and 5.8% of the control group, revealing for the small animal veterinarians an increased risk for exposure to this virus. Besides NoVs, the zoonotic potential is also suspected for other caliciviruses as the novel 2117-like vesiviruses (VeVs), firstly identified in dog stool samples in Italy [188]. IgG antibodies against the canine 2117-like VeVs have been detected in 7.8% of Italian human sera [189] and, more recently, the RNA of a 2117-like VeV was detected in the feces of a clinically healthy cat [190]. Accordingly, understanding the ecology of novel enteric viruses in cats will be helpful also to assess more precisely if and to which extent pets may pose a risk of infection for humans.

## Figures and Tables

**Table 1:** Novel viruses detected in the stool samples of cats by the molecular approach since 2012.

Family	Viruses	Genome	Country	Year	Molecular Tools	Target Gene	References
*Caliciviridae*	Feline norovirus	ssRNA	USA	2012	RT-PCR: consensus primers	RdRp	[11]
*Parvoviridae*	Feline bocavirus	ssDNA	China	2011	PCR: consensus primers	NS1	[12]
*Picornaviridae*	Feline kobuvirus	ssRNA	South Korea	2013	RT-PCR: broadly reactive primers	RdRp	[13]
*Parvoviridae*	Feline bufavirus	ssDNA	Italy	2016	PCR: broadly reactive primers	VP2	[14]
*Circoviridae*	Feline stool-associated circular DNA virus	ssDNA	Japan	2018	Nested-PCR: consensus primer	Rep	[15]
	Feline cyclovirus		USA	2013	Metagenomic sequencing		[17]
*Picornaviridae*	Feline sakobuvirus A	ssRNA	Portugal	2012	Metagenomic sequencing		[16]
*Reoviridae*	Rotavirus I *Felis catus*	dsRNA	USA	2016	Metagenomic sequencing		[18]
*Polyomaviridae*	Lyon-IARC polyomavirus	dsDNA	Canada	2019	Metagenomic sequencing		[19]

**Table 2:** List of primers used for detection of feline NoVs.

Primer	Sequence (5′ to 3′)	Sense	Target	References
p289 p290	TGACAATGTAATCATCACCATA GATTACTCCAAGTGGGACTCCAC	+ -	RdRp	[54]
JV12Y JV13I	ATACCACCTATGATGCAGAYTA TCATCATCACCATAGAAGAG	+ -	RdRp	[55]
FNoV-F9d FNoV-R15	GCCCACTGGATWTACACCCTCTC CTGATGGTTGGGTCCTCTGGTCCA	+ -	RdRp	[11]
FNoV-F9d FNoV-R14d	GCCCACTGGATWTACACCCTCTC CYTGGT RTACCCAAACTCCA C	+ -	ORF2	[11]
QT	CCAGTGAGCAGAGTGACGAGGACTCGAGCTCAAGC (T^17^)	+/-	3′/5′end	[56]

**Table 3:** Molecular studies detecting FeKoV in cats. Prevalence in diarrheic and non-diarrheic samples are reported.

Country	Samples Tested			Positive Samples			References
	Total	Diarrhoeic	Non Diarrhoeic	Total	Diarrhoeic	Non Diarrhoeic	
**Korea**	39	39	0	6 (15.4%)	6 (15.4%)	0	[13]
**Korea**	71	52	19	17 (23.9%)	15 (28.8%)	2 (10.5%)	[90]
**Italy**	83	37	46	5 (6.0%)	5 (13.5%)	0 (0.0%)	[85]
**China**	81	52	29	8 (9.9%)	8 (15.4%)	0 (0.0%)	[86]
**China**	197	105	92	28 (14.2%)	20 (19.1%)	8 (8.7%)	[87]

**Table 4:** Novel parvoviruses identified in cats and their current classification.

Genus	Species	Common Names Used in Literature	Country	Year	Detection Source	References
*Bocaparvovirus*	*Carnivore bocaparvovirus 3*	Feline bocaparvovirus (FBoV)	China	2011	Stools, urine, kidney, blood and respiratory samples	[12]
	*Carnivore bocaparvovirus 4*	FBoV2	Portugal	2012	Stools	[16]
	*Carnivore bocaparvovirus 5*	FBoV3	USA	2013	Stools	[17]
*Protoparvovirus*	*Carnivore protoparvovirus 2* ^*^	Feline bufavirus (FBuV)	Italy	2017	Stools and respiratory samples	[14]

* Novel candidate species.

**Table 5:** List of consensus or specific primers used for the detection of FBoVs.

Genus/Species	Primer	Nucleotide Sequence (5′to 3′)	Lenght (bp)	Target	References
BoVs	BoVF	GCCAGCACNGGNAARACMAA	141	NS1	[12]
	BoVR	CATNAGNCAYTCYTCCCACCA			
FBoV	FBoV1F	TCTACAAGTGGGACATTGGA	133	NS1	[12]
	FBoV1R	GAGCTTGATTGCATTCACGA			
FBoV2	FBoV2F	TCGTTCGTCTTGGAACATAGC	301	NS1	[16]
	FBoV2R	CAGAGCGTGGATCTGTCTGA			
FBoV3	FBD1L1	TGACTCGTCTGTGGCGGGCT	546	VP1	[17]
	FBD1R1	TCGTTCGTGAGACGCTGCCA			
	FBD1L2	CAAAGGATCGGGAGCGGGCG	388	VP1	[17]
	FBD1R2	TGCCCATGGTGTTGTGATTCCTATCCA			
